# Nitrogen deposition may enhance soil carbon storage via change of soil respiration dynamic during a spring freeze-thaw cycle period

**DOI:** 10.1038/srep29134

**Published:** 2016-06-30

**Authors:** Guoyong Yan, Yajuan Xing, Lijian Xu, Jianyu Wang, Wei Meng, Qinggui Wang, Jinghua Yu, Zhi Zhang, Zhidong Wang, Siling Jiang, Boqi Liu, Shijie Han

**Affiliations:** 1 College of Agricultural Resource and Environment, Heilongjiang University, 74 Xuefu Road, Harbin, 150080, China; 2 School of forestry, Northeast Forestry University, 26 Hexing Road, Harbin, 150040, China; 3 Institute of forestry science of Heilongjiang province, 134 Haping Road, Harbin 150081, China; 4 Institute of Applied Ecology, Chinese Academy of Sciences, 72 Wenhua Road, Shenyang, 110016, China

## Abstract

As crucial terrestrial ecosystems, temperate forests play an important role in global soil carbon dioxide flux, and this process can be sensitive to atmospheric nitrogen deposition. It is often reported that the nitrogen addition induces a change in soil carbon dioxide emission in growing season. However, the important effects of interactions between nitrogen deposition and the freeze-thaw-cycle have never been investigated. Here we show nitrogen deposition delays spikes of soil respiration and weaken soil respiration. We found the nitrogen addition, time and nitrogen addition×time exerted the negative impact on the soil respiration of spring freeze-thaw periods due to delay of spikes and inhibition of soil respiration (*p* < 0.001). The values of soil respiration were decreased by 6% (low-nitrogen), 39% (medium-nitrogen) and 36% (high-nitrogen) compared with the control. And the decrease values of soil respiration under medium- and high-nitrogen treatments during spring freeze-thaw-cycle period in temperate forest would be approximately equivalent to 1% of global annual C emissions. Therefore, we show interactions between nitrogen deposition and freeze-thaw-cycle in temperate forest ecosystems are important to predict global carbon emissions and sequestrations. We anticipate our finding to be a starting point for more sophisticated prediction of soil respirations in temperate forests ecosystems.

Carbon (C) cycles are increasingly paid attention under global climate change. Freeze-thaw-cycle (FTC) significantly affects soil C cycles as a crucial ecological process^1^,^2^,^3^ due to its more frequent appearance under global climate change^4^,^5^,^6^. Therefore FTC is recognized as crucial ecological processes and has received increased attention. Studying on the impacts of FTC on soil C dynamic is beneficial to the further understanding of soil C cycle and their feedback to climate change.

Many previous studies have pointed out that the FTC-induced enhancement of carbon dioxide (CO_2_) emission was often observed^7^,^8^,^9^,^10^,^11^,^12^,^13^,^14^,^15^,^16^,^17^,^18^. Wang *et al*.^16^ showed that the ephemeral burst of CO_2_ occurred at the early stage of spring FTC period in a temperate forest. Song *et al*.^15^ found the high emission peaks of CO_2_ during FTC period in a freshwater marsh. Wang *et al*.^17^ suggested that FTC play an important role in soil CO_2_ emissions in a wet meadow. In addition, the CO_2_ emission peaks during the FTC period were also detected in some laboratory incubations^10^,^19^, which are consistent with most of the field studies. However, different conclusions have been also reported. For example, the FTC had no a significantly impact on CO_2_ emission in broadleaf forests or it reduced the release of CO_2_ in grassland^20^,^21^. The emission of CO_2_ from soil is one of major C exchanges between terrestrial ecosystems and the atmosphere^22^. With global climate change, less snowfall and warming may lead to increasing the frequency and intensity of FTC, and then may cause the increase of CO_2_ emission from soil to atmosphere. Sullivan *et al*.^23^ suggested that the pulses of CO_2_ caused by FTC are jointly driven by biological and physical factors. Several potential mechanisms have been proposed to clarify the FTC-induced enhancement of CO_2_ emissions: (1) burst of CO_2_ during the FTC period largely resulted from the release of trapped CO_2_ in the winter^7^; (2) increased CO_2_ emissions may be due to enhancing microbial metabolism by substrate supply in the FTC period^21^; (3) increased substrates leaching from the litter layer accumulated during the winter might lead to CO_2_ burst^24^. Currently, there are still many uncertainties in the mechanisms of these increased CO_2_ fluxes^25^. The first objective of our study was to examine the impact of spring FTC on the soil CO_2_ emissions in the temperate forest, and then to investigate the mechanisms potentially inducing FTC period CO_2_ emissions.

In addition, atmospheric nitrogen (N) deposition is another important factor to soil C cycle, because the cycles of soil C and N are closely coupled^26^,^27^,^28^. Some previous studies showed that simulated N addition had significantly increase release of CO_2_ 29. Nevertheless, other studies found controversial affecting soil CO_2_.fluxes in terrestrial ecosystem^30^,^31^. The different responses of soil CO_2_ fluxes to N addition have been reported in the different ecosystem, including increases^26^, decreases^32^, and no significant differences^33^,^34^. Summary, most of the high concentration N deposition may limit CO_2_ release, and low concentration may promote or no changes. Several potential mechanisms have been proposed to clarify the N-induced change of CO_2_ emissions: (1) N inhibition of lignin degradation largely resulted from change of microbial composition^35^; (2) change of CO_2_ emissions may be due to ecological shifts in the soil microbiota under N deposition^36^; (3) the coupling of soil carbon and nitrogen was broken due to N deposition, which might lead to change of CO_2_ emission^37^. Although the effect of FTC on C cycles and the effect of atmospheric N deposition on C cycles have been investigated, respectively^28^,^38^,^39^, the effect of FTC together with atmospheric N deposition on C cycles has never been reported. We hypothesized that soil respiration (*R*s) could have a special response pattern to N deposition due to the changes of soil physicochemical properties and microbial characteristic in the FTC period. The second objective of our study was to examine the impact of simulated N deposition together with spring FTC on soil CO_2_ fluxes in temperate forest.

The major objective of this paper was to evaluate the change quantities of CO_2_ due to N deposition addition in FTC period in temperate forests which cover 9.7% of the earth’s continental surface^40^. We hypothesized that N deposition would inhibit CO_2_ emissions via delay burst or decrease fluxes in spring FTC period in temperate forest. In addition, previous studies did not show an understandable mechanism regarding impact of FTC and N deposition on CO_2_ fluxes. The FTC and N deposition could affect the soil biological and physicochemical processes leading to C dynamic change. Therefore, we conducted a simulated N deposition experiment from May 2010 to present, and investigated the interactive effects of N deposition and spring FTC on soil C fluxes in a temperate forest and the potential mechanisms in 2015.

## Materials and Methods

### Site description

This study was conducted at the Fenglin Natural Reserve of Lesser Khingan Mountains in Heilongjiang province, Northeast China (48°02′–48°12′ N, 128°58′–129°15′ E). The climate is continental monsoon climate, with dry, cold winters, and humid, warm summers. The forests had a mean annual temperature of −0.5 °C from 1959 to 2013, with the lowest and highest monthly mean air temperature being −25.6 °C in January to 23.8 in July, respectively. The mean annual precipitation is 728 mm, of which approximately 75% falls between July and August. The snowpack lasted for 148 days with the snow depth ranging from 0 to 42 cm during the measurement years (Nov, 2014–Mar, 2015). The soil is classified as a dark brown forest soil^41^. The vegetation type is a cold-temperate spruce-fir Korean pine forest with the age of exceeded 200 years. The community is dominated by *Picea koraiensis*, *Abies nephrolepis* and *Pinus koraiensis*. The mean stand density is 972 ± 96 trees ha^−1^, the mean diameter at breast height is 13.7 ± 7.5 cm and the mean tree height is 16.7 ± 5.3 m. The major species in the canopy layer are *Pinus koraiensis, Abies nephrolepis, Picea koraiensis, Picea jezoensis var. microsperma, Larix gmelini, Betula platyphylla, Acer mono, Fraxinus mandshurica* and *Betula costata*.

### Experimental design

To investigate changes in soil CO_2_ fluxes (*R*s) following N application, we established three random blocks in May 2010, and each consisting of four research plots measuring 20 m × 20 m. The plots were separated by 10 m wide buffer strips to avoid horizontal movement of the soil N. The simulated N deposition was initiated at the onset of this experiment and included four treatments, control (no added N), low-N (5 g N.m^−2^.yr^−1^), medium-N (10 g N.m^−2^.yr^−1^) and high-N (15 g N.m^−2^.yr^−1^), with three replicates randomly distributed at each treatment. The N was applied as ammonium nitrate (NH_4_NO_3_) solution and was distributed on six occasions during annual growing season applied to the forest floor every half a month during the growing season (May to October) from 15th May 2010. In each plot, the NH_4_NO_3_ was mixed with 32 L of water (equal to 0.08 mm annual precipitation), and applied by using a backpack sprayer below the canopy. Two passes were made across each plot to ensure an even distribution of the fertilizer. The control plots received 32 L water without N addition. The simulated N deposition was applied from May 2010 to the present.

### Soil CO_2_ fluxes measurements during spring FTC period

The spring FTC period soil CO_2_ fluxes (*R*s) were measured every day from April 1^st^ to May 5^th^ 2015. For each of 12 plots, three polyvinyl Chloride (PVC) collars (20 cm inside diameter and 12 cm in height) were randomly inserted approximately 9 cm into the soil, with 3 cm left above the ground surface for *R*s measurements, one week before N addition in 2010. A total of 36 soil collars were installed. The collars were left in the same place throughout the entire study period for exploring the change of the spring FTC period in *R*s. The *R*s was measured with a Li-8100 automated soil CO_2_ flux system (Li-Cor Inc, Lincoln, NE, USA) between 10:00–14:00 in spring FTC period. Each measurement was repeated 3 times for each collar to produce a collar’s mean *R*s rate. *R*s were calculated using exponential regression model with the LI-8100 file viewer application software (LI-8100/8150 Instruction Manual).

### Soil physical and chemical properties and microbial characteristic measurements

The soil temperature at 5 cm depth (*T*_5_) and soil volumetric water content at the 5 cm depth (*W*_5_, % v/v) were monitored simultaneously with the measurement of *R*s by using a soil temperature probe (Omega Engineering Inc. USA) and soil moisture probes (Deltat Devices Ltd., Cambridge, England) connected to Li-8100. The continuous soil temperature at 5 cm depth (*T*_5cm_) was monitored hourly by Em-50 data logger (Decagon Devices, Inc. USA) The air temperature (*T*_a_) was same measured hourly by Em-50 data logger (Decagon Devices, Inc. USA).

During the measurement of *R*s, because of the difficulty in collecting soil samples from frozen soil, all soil samples were collected days nearly the soil collars from a depth of 0–10 cm using a specially designed auger (2.5 cm in diameter). Three soil cores were collected and pooled to one composite sample at each plot. All of the visible extraneous materials (such as roots, stones, etc.) were removed by hand, and then divided the composite sample into three sub-samples. One sub-sample was air-dried at ambient temperature, and then sieved (2 mm) and ground for the analysis of soil total C and total N by using an automated TOC/TN analyzer (multi N/C3100, Analytikjene AG, Germany). In addition, soil pH values were measured by a pH meter (SX7150, China) with soil: water ratio of 1:2.5. The second sub-sample was maintained original state, and taken back to laboratory. Thawed soils were mixed, whereas frozen soil was reduced to small pieces, with the pieces being homogenized to the extent possible^42^. Immediately following, the inorganic N concentrations were determined by extracting fresh soil with K_2_SO_4_. The extractable NH_4_^+^-N concentrations were measured by using the indophenol blue method, followed by the colorimetric analysis. The NO_3_^−^-N content was determined by using the copper-cadmium reduction method. The third sub-sample was also maintained original states, and taken back to the laboratory immediately to assess microbial biomass C (MBC) and N (MBN). The MBC and MBN were measured by using a fumigation-extraction method^43^. The extracts of N and C from fumigated and unfumigated samples were analysed by an automated TOC/TN analyzer (multi N/C3100, Analytikjene AG, Germany). The MBN and MBC were calculated from the difference between extractable N and C contents in the fumigated and the unfumigated samples using conversion factors (*k*EN and *k*EC) of 0.45 and 0.38, respectively^43^. All extraction for NO_3_-N, NH_4_-N, MBC and MBN was done with K_2_SO_4_ of 0.5 mol l^−1^ in 25 °C, and the duration of extraction was half an hour.

### Dividing the year into spring FTC period and Statistical analyses

The spring FTC period was defined as the period that starts when soil surface snow is start to melt (the maximum *T*_a_ is above 0 °C) and ends when daily minimum *T*_5cm_ is above 0 °C^16^. The spring FTC period lasted for 35 days (DOY 90–124 in 2015) in this study.

To assess the quantity of *R*s under different N addition level in the FTC periods, *R*s-*T* models were constructed. Compared to the several commonly used models, such as the modified van’t Hoff’s model^44^, the sigmoid-shaped Lloyd-Taylor^45^ and logistic models^46^, the Gamma model performed either better or as good as the other models^47^. In addition, Gamma model were tested across a wide *T*s range (−18–35 °C) and can also be expanded, using simple mathematics to help researchers analyse the *R*s-*T* relationship in the context of other environment factors, such as soil nutrients^47^. The Gamma model was adopted based on R^2^ and the Akaike Information Criterion (AIC). Therefore, Gamma model used to assess the impact of different quantities of N additions on *R*s during the FTC period.

Gamma model was expressed as following:


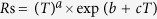


where T is (T_5cm_ + 40), a, b and c are regression coefficients. T_5cm_ is measured soil temperature under 5 cm below surface. 40 °C is added to T_5cm_ because negative T_5cm_ results in negative or imaginary *R*s (or non-meaningful *R*s), and 40 °C has been chosen as the lowest T_5cm_ where *R*s continues has been measured at −39 °C. The natural logarithm (Ln) transformed version of the *R*s data was applied to alleviate the heteroscedasticity problem.

Two-ways analysis of variance was used to examine the impacts of different quantities N deposition, spring FTC and their interactions on soil total C, total N, NH_4_^+^-N, NO_3_^−^-N, soil pH values, MBC, MBN. Fisher’s LSD followed the two-way analysis of variance between the N treatments. Tukey’s HSD tests were used to reveal the significant pairwise differences of the N addition. Pearson’s correlation analysis was used to determine the correlations between *R*s and soil properties or microbial characteristics. Statistically significant differences were accepted at p < 0.05. All statistical analyses were performed using R 3.2.2 Version Software (R Development Core Team 2015).

## Results

### Effects of spring FTC, N deposition and their interaction on *R*s

**Figure 1:**
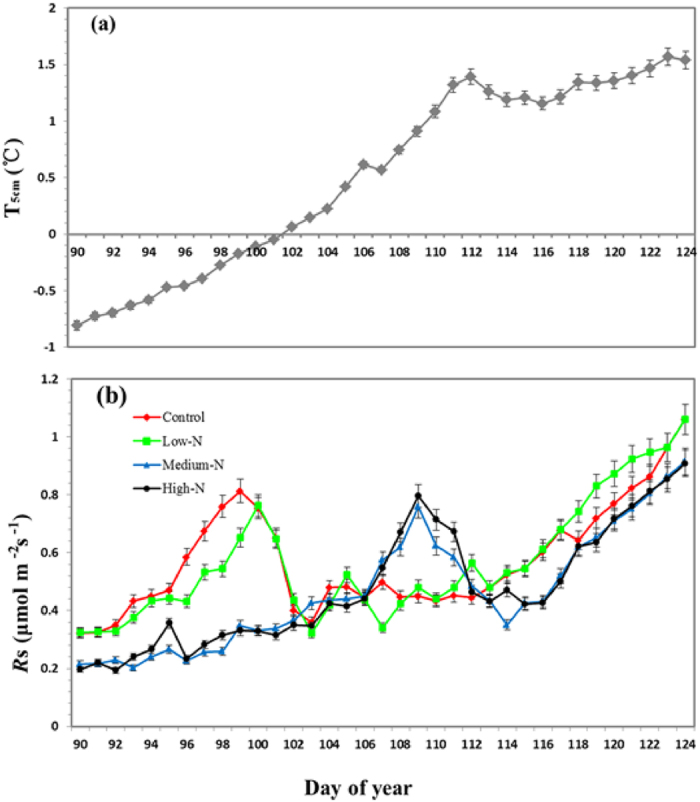
(**a**) Mean daily variation of soil temperature at the 5 cm depth (T_5cm_) during the spring Freeze-thaw cycle periods in 2015. (**b**) Daily variation of soil respiration at different added N level plots in spring Freeze-thaw cycle periods in 2015. Control refers the control treatment plots; Low-N refers the Low-N treatment plots; Medium-N represents the Medium-N treatment plots; High-N represents the High-N treatment plots.

At the beginning of the spring FTC period, the daily maximum *T*_a_ was above 0 °C, but all of *T*_5cm_ were below 0 °C (Fig. 1(a)), and the snow was melting. However, the *R*s remained at a low level (Fig. 1(b)), and the *R*s under medium-N and high-N treatments was significantly lower than control and low-N treatments at the early stage of the spring FTC period (Fig. 1(b)). The significant differences in *R*s were observed on next period of time, and temporal peaks of *R*s occurred. The ephemeral burst of *R*s observed from DOY 97 to DOY 102 under control treatments and lasted for 6 days, with the maximum *R*s of 0.83 μmol m^−2^ s^−1^ (Fig. 1(b)). Simultaneously, we observed the high *R*s occurred from DOY 98 to DOY 102 under low-N treatments and lasted for 5 days, with the maximum *R*s of 0.76 μmol m^−2^ s^−1^ (Fig. 1(b)). During the period, the daily mean of air temperature and the mean of soil temperature in 5 cm depth increased continuously (Fig. 1(a)). The snowpack had melted completely. But, the ephemeral enhancement of *R*s occurred at later stage of the spring FTC and lasted for 5 days (DOY 107–111) under medium-N and high-N treatments (Fig. 1(b)). The *R*s pulse lasted for a short time period and after that the rate decreased to the normal status during the spring FTC period. During most of observation period, the *R*s increased with temperature.

The effects of different quantity of N addition on *R*s were highly variable during spring FTC period. During the measurement period, the mean of *R*s was 0.58, 0.57, 0.47, 0.48 μmol m^−2^ s^−1^ for different quantity of N addition, i.e., control, low-N, medium-N, high-N treatments, respectively (Table 1). Our results found that the simulated N deposition had significantly impact on the *R*s due to inhibiting CO_2_ fluxes or delaying outburst event (Table 1; Fig. 1(b)). Likewise, the FTC also had a significantly impact on *R*s (Table 2), which varied from 0.32 to 1.06 μmol m^−2^ s^−1^ and showed the high fluctuations under natural status (control plots) (Fig. 1(b)). In addition, *Rs* was also significantly affected by the interaction of the simulated N deposition and spring FTC (p < 0.001) (Table 2). In general, the two-way ANOVA analysis showed that the simulated N deposition, the spring FTC and their interaction exhibited significant effects (*p* < 0.001) on the *R*s during the whole measurement period (Table 2).

### Spring FTC period contribution of *R*s to the winter and annual budget and assessing the future C dynamic in temperate forest

**Figure 2:**
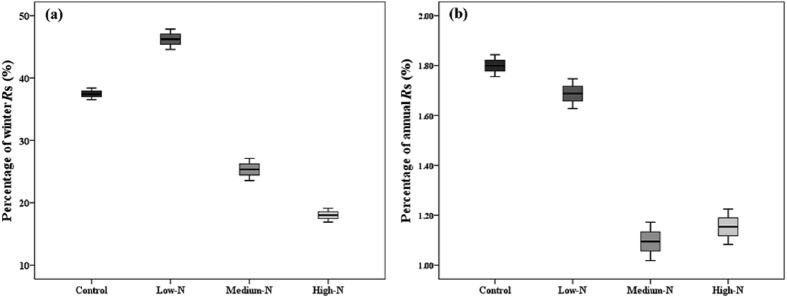
Model-based contributions of spring Freeze-thaw cycle soil CO_2_ flux to winter (**a**) and annual total (**b**) at the different quantities of N addition (control, Low-N, Medium-N, High-N). The winter and annual soil CO_2_ efflux quote from Liu *et al*.^48^.

Applying the empirical *R*s-T models assessed the quantities of *R*s under different N addition levels (i.e., control, low-N, medium-N, high-N) during the spring FTC period. The ordinary least squares was used to calculate the coefficients (i.e., a, b, c), similar to what was performed in the Khomik 2009 Gamma model paper (Table 3). The predicated spring FTC period *R*s was 17.53 ± 0.43 g C m^−2^ yr^−1^ in this temperate forest without N addition (Table 1). Low-N treatment exerted negative effects on spring FTC *R*s, and its value was 16.44 ± 0.58 g C m^−2^ yr^−1^ (Table 1). The cumulative *R*s during the spring FTC period were 10.67 ± 0.75 g C m^−2^ yr^−1^ in medium-N plots and 11.24 ± 0.69 g C m^−2^ yr^−1^ in high-N plots (Table 1). In general, the N addition exerted a negative impact on spring FTC *R*s and decreased it by 6% (low-N), 39% (medium-N) and 36% (high-N) compared with the control. The predicted annual *R*s was 974.3 ± 67.1 g C m^−2^ yr^−1^ without N addition treatment; the values of *R*s in winter were 46.8 g C m^−2^ yr^−1^ (control), 35.7 g C m^−2^ yr^−1^ (low-N), 41.89 g C m^−2^ yr^−1^ (medium-N) and 62.35 g C m^−2^ yr^−1^ (high-N)^48^. Under different quantities of N addition, the cumulative *R*s during spring FTC period contributed 37.49% (control), 46.88% (low-N), 25.50% (medium-N) and 18.03% (high-N), respectively, to the winter *R*s and contributed 1.80% (control), 1.69% (low-N), 1.10% (medium-N) and 1.15% (high-N), respectively, to the annual *R*s (Fig. 2).

The Fenglin Natural Reserve (our study site) covered an area of 18165 hm^2^. We hypothesized that whole Reserve was used to simulate the impact of N addition on *R*s. The *R*s in the study area was reduced by 1.97 × 10^−4^ Tg C yr^−1^ (low-N), 1.25 × 10^−3^ Tg C yr^−1^ (medium-N) and 1.14 × 10^−3^ Tg C yr^−1^ (high-N) during the spring FTC period. The temperate forest covers 9.7% of the earth’s continental surface^40^. The temperate forest covered an area of 14.5 million km^2^. The *R*s in whole temperate forest would be reduced by 15.81 Tg C yr^−1^ (low-N), 99.47 Tg C yr^−1^ (medium-N) and 91.21 Tg C yr^−1^ (high-N) during the spring FTC period. Global total CO_2_ emission (excluding Land-use Change and Forestry) cumulative value was 33843.05 Mt (about 9229.92 Tg C) in 2012 49. The decrease values of *R*s under medium- and high-N treatments during spring FTC period in temperate forest would be approximately equivalent to 1% of global annual C emissions.

### Relationships between spring FTC *R*s and soil biochemical property

**Figure 3:**
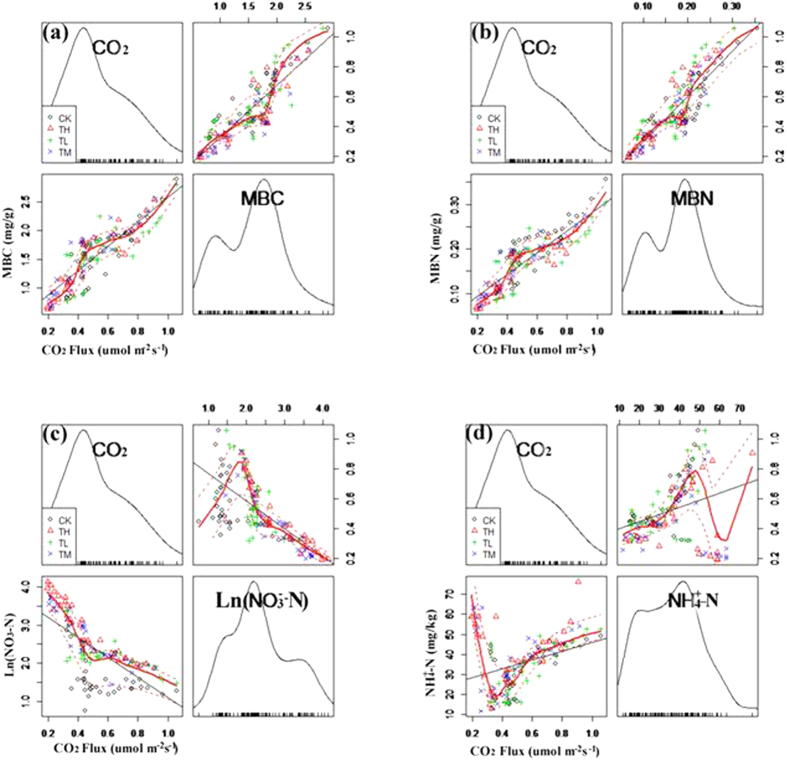
Relationships between (**a**) the soil CO_2_ efflux and microbial biomass carbon (MBC, R^2^ = 0.75), (**b**) the soil CO_2_ efflux and microbial biomass nitrogen (MBN, R^2^ = 0.74), (**c**) the soil CO_2_ efflux and nitrate nitrogen concentrations (NH4^+^-N, R^2^ = 0.63), (**d**) the soil CO_2_ efflux and ammonium nitrogen concentrations (NO_3_^−^-N, R^2^ = 0.33) at the different quantities of N addition (CK refers the control treatment plots; T_L_ refers the Low-N treatment plots; T_M_ represents the Medium-N treatment plots; T_H_ represents the High-N treatment plots) during the spring Freeze-thaw cycle periods. Pictures made by Lattice package (R 3.2.2 Version).

The mean values of soil biochemical property were summarized in Table 4. The correlation analyses between *R*s and soil biochemical property were performed to attempt to explain the observed changes in *R*s during spring FTC period. But we only found the *R*s and the soil NH_4_^+^-N, the soil NO_3_^−^-N, the soil MBC and the soil MBN are related. The *R*s was positively correlated with the soil MBC and MBN during the spring FTC period (Fig. 3a,b). But, the *Rs* was positively correlated with the lower concentrations of the soil NH_4_^+^-N and the soil NO_3_^−^-N, and negatively correlated with the higher concentrations of the soil NH_4_^+^-N and the soil NO_3_^−^-N during spring FTC period (Fig. 3c,d). We made the best fitting equation of the *R*s and the soil biochemical property. The *R*s increased linearly with the soil MBC (y = 0.47x − 0.21, R^2^ = 0.75, *p* < 0.01, y was defined as *R*s values, x was defined as MBC values) and the soil MBN (y = 4.07x − 1.83, R^2^ = 0.74, *p* < 0.01, y was defined as *R*s values, x was defined as MBN values). The *R*s decreased exponentially with the soil NO_3_^−^-N concentrations (y = 0.70e^−0.03x^, R^2^ = 0.63, *p *< 0.01, y was defined as *R*s values, x was defined as soil NO_3_^−^-N concentrations). The *R*s changed irregularly with the soil NH_4_^+^-N concentrations (y = 139.55x^2^−139.89x + 64.82, R^2^ = 0.33, *p* < 0.05, y was defined as *R*s values, x was defined as soil NH_4_^+^-N concentrations).

## Discussion

We found that the ephemeral spikes of *R*s occurred in control plots (without N addition) at the early stage of the spring FTC period, which was consistent with some previous observation^16^,^19^,^25^. At the beginning of the outburst, the snow was completely melted. And, the soil microbes can recover rapidly from disturbance resulting from freezing^50^. Therefore, we conjectured that microbial activity and biomass may increase via enhanced substrate supply and available water (liquid water) result in *R*s emission pulses. Wang *et al*.^16^ also suspected that *R*s pulses might be related to the soil hydrology changes in the spring FTC period. Priemé and Christensen^51^ pointed out that the mechanisms for *R*s pulses during the FTC period were stimulated by microbial metabolism via the enhanced substrate supply, which was also consistent with our results. During the outburst period, we measured the MBC and the MBN that were significantly difference among all treatments (Table 4), and the results supported our conjecture. However, the rate of *R*s gradually decreased to a normal level after short time pulses. We considered that the soil microbial activity or biomass were higher in the early stage of spring FTC and decreased in the following cycles, indicating that successive FTCs might lead to the decrease of the microbial biomass in the soil examined. There are some consistent explanations for the phenomenon^10^,^51^,^52^. Schimel and Clein’s^53^ study shown that the successive FTCs might lead to the lysis of microbial cells, and followed by the decrease of *R*s. In addition, Haei *et al*.^54^ suggested that the change of dissolved organic carbon (DOC) might also impact on *R*s during FTCs, and the decreased rate of *R*s after the pulses could be due to change of DOC utilization.

We also found the nitrogen addition exerted the negative impact on the soil respiration of spring freeze-thaw periods due to delay of spikes and inhibition of soil respiration. The mechanisms for impact of N addition on *R*s during the spring FTC period are complicated. In our study, the mechanism for FTC-induced enhancement of *R*s is not consistent with the conjecture of Elberling and Brandt^7^ that a pulse during the FTC period resulted from the release of trapped CO_2_ in the winter. We suggested that a relatively high microbial biomass is more likely to release a pulse of CO_2_ during FTC than a relatively low microbial biomass. With respect to the delay under N addition, we hypothesized N and salt in high concentrations inhibited microbial activity and biomass during the early period of FTC so that pulse of *R*s did not occur in this period. After this period of FTC, the pulse of *R*s was observed, because the continuous FTC promoted N leaching losses^55^, which decreased the inhibition of microbial activity and biomass. Simultaneously, when most of the extrinsic inhibitor can be removed, the microbial activity and the biomass may rapidly increase resulting in the *R*s emission pulses in treatments plots. The hypothesis is also supported by the results of others^56^,^57^,^58^,^59^,^60^,^61^.

The contributions of *R*s during the spring FTC period to the annual *R*s were 1.80%, 1.69%, 1.10% and 1.15% for control, low-N, medium-N and high-N treatment, respectively (Table 1, Fig. 3). Our results suggested that response of *R*s to simulated N deposition in temperate forests is a decline, and it may vary depending on the level of N deposition during the spring FTC periods. In the previous studies, the decrease in *R*s occurred in the warm and wet growing season in the N addition plots^62^,^63^,^64^, and not occurred in winter among treatment^63^. In addition, our results also suggested that contribution of *R*s during the spring FTC period to the annual *R*s will vary when the global N deposition are greatly altered with the atmospheric N levels rise^64^. We suspected that the decline of *R*s due to N addition may be an improvement in the C use efficiency of the soil microbial community, and might impact on the global C cycle. However, N deposition may enhance soil carbon storage via decrease of *R*s during spring FTC period. Therefore, more attention should be paid to the impact of N deposition on soil respiration in the spring FTC period.

## Conclusions

The simulated N addition delayed the outburst of *R*s compared with control (no N addition). The soil spring FTC decreased the soil C that releases into atmosphere under N deposition. The relative diminution of *R*s induced by N addition may potentially affect C cycle in temperate forest. In general, the effects of N addition and spring FTC on *R*s are very important to accurately predict soil CO_2_ flux in cold region forest ecosystems under a changing climate.

## Additional Information

**How to cite this article**: Yan, G. *et al*. Nitrogen deposition may enhance soil carbon storage via change of soil respiration dynamic during a spring freeze-thaw cycle period. *Sci. Rep*. **6**, 29134; doi: 10.1038/srep29134 (2016).

## Figures and Tables

**Table 1:** Spring FTC periods soil CO_2_ flux and contribution of *R*s to the winter and annual budget at the different quantities of nitrogen additions treatments.

Specified Treatments	*R*s (umol CO_2_ m^−2^ s^−1^)	Cumulative *R*s (g C m^−2^)	Contribution to winter *R*s (%)	Contribution to annual *R*s (%)
Control-N	0.58 ± 0.02	17.53 ± 0.43	37.49 ± 0.89	1.80 ± 0.02
Low-N	0.57 ± 0.01	16.44 ± 0.58	46.88 ± 1.32	1.69 ± 0.01
Medium-N	0.47 ± 0.02	10.67 ± 0.75	25.50 ± 1.47	1.10 ± 0.01
High-N	0.48 ± 0.03	11.24 ± 0.69	18.03 ± 0.85	1.15 ± 0.01

**Table 2:** ANOVA *P*-Values for impact of Treatment and FTC (Substitute Date for FTC) on soil respiration (*R*s), microbial biomass carbon (MBC), microbial biomass nitrogen (MBN), ammonium nitrogen concentrations (NH_4_^+^-N), nitrate nitrogen concentrations (NO_3_^−^-N).

Effect	*R*s	MBC	MBN	NO_3_^−^-N	NH_4_^+^-N
Treatment	<0.001	<0.001	<0.001	<0.001	<0.001
Date	<0.001	<0.001	<0.001	<0.001	<0.001
Treatment × Date	<0.001	<0.001	<0.001	<0.001	<0.001

*P*-Values < 0.001 denote very significance.

**Table 3:** Regression models of *R*s against soil temperature at the 5 cm depth (*T*_5_) for the FTC period.

Treatments	a	b	c	R^2^	AIC
Control	−220.01	585.95	5.62	0.61	−64
Low-N	−480.62	1285.47	12.17	0.43	−48
Medium-N	424.54	−1166.35	−10.02	0.80	−79
High-N	366.40	−1009.17	−8.59	0.81	−84

The regression models are of the form: *Rs* = *T*^*a*^ × exp (*b* + *cT*), where *T* is (*T*_5_ + 40), a, b and c are regression coefficients, and determination coefficient(R^2^) and Akaike Information Criterion (AIC) are given.

**Table 4:** The mean values of Soil NH_4_^+^-N, Soil NO_3_^−^-N, Soil Microbial C, Soil Microbial N, Soil Total C, Soil Total N and soil pH during the spring FTC period.

	Control-N	Low-N	Medium-N	High-N
Soil NH_4_^+^-N(mg/kg)	5.74 ± 4.64d	10.29 ± 5.55c	18.86 ± 12.19b	22.24 ± 16.27a
Soil NO_3_^−^-N(mg/kg)	32.48 ± 11.02c	32.47 ± 11.99d	38.31 ± 15.04b	39.12 ± 14.88a
Soil Microbial C(mg/g)	1.64 ± 0.46a	1.63 ± 0.47b	1.49 ± 0.51c	1.47 ± 0.51d
Soil Microbial N(mg/g)	0.20 ± 0.05a	0.18 ± 0.05b	0.16 ± 0.06c	0.15 ± 0.05d
Soil Total C(mg/g)	103.10 ± 5.13b	169.32 ± 6.45a	171.85 ± 4.82a	166.63 ± 7.21a
Soil Total N(mg/g)	13.17 ± 3.28b	15.43 ± 5.86b	16.19 ± 7.46a	19.29 ± 6.82a
Soil pH	5.10 ± 0.48a	5.00 ± 0.57a	4.87 ± 0.61b	4.72 ± 0.75b

Value within the same column with the same letters (a, b, c and d) are not significantly different at *p* < 0.05. Data are shown as means with standard errors.
